# How Does Social Behavior Relate to Both Grades and Achievement Scores?

**DOI:** 10.3389/fpsyg.2018.00857

**Published:** 2018-06-04

**Authors:** Jeffrey M. DeVries, Katharina Rathmann, Markus Gebhardt

**Affiliations:** Faculty of Rehabilitation Sciences, Technische Universität Dortmund, Dortmund, Germany

**Keywords:** prosocial behavior, peer problems, grades, competency, large-scale assessment, structural equation modeling, academic achievement

## Abstract

Prosocial behavior and peer problems are an important correlate of academic development; however, these effects vary by achievement measures and social behaviors. In this paper, we examined data from the German National Education Panel Study (NEPS), and we use structural equation modeling (SEM) to model the effects of prosocial behavior and peer problems on grades and competencies for both math (*n* = 3,310) and reading (*n* = 3,308) in grades 5 and 7. Our models account for the moderating effect of both gender and socioeconomic status (SES) as determined by parental education. We conclude that social behaviors relate to grades more strongly than competencies, that peer problems relate more strongly to achievement than prosocial behavior, and that the relationship is weaker in later grades. We discuss the implication that grades and achievement tests are not interchangeable measures for educators and researchers.

## Introduction

Academic progress can be measured in multiple ways including grades and achievement scores, but these methods are not interchangeable. Grades are more strongly connected to multiple noncognitive factors, including social behaviors, than achievement tests (Borghans et al., 2011; Farrington et al., 2012; Lechner et al., 2017). Although social behaviors are an indirect predictor, they can broadly predict future academic success (Durlak et al., 2010). However, due to their indirect nature, sufficiently large-scale studies are required to discern the differential relationship social behaviors have with both grades and achievement scores. The National Education Panel Study (NEPS; Blossfeld et al., 2011) is a large-scale longitudinal study of multiple cohorts of German students, which gives a unique opportunity to examine such relationships. In this paper, we model the relationship between social behaviors (specifically prosocial behavior and peer problems), competency, and grades with data from NEPS, in order to unravel which academic measures (grades vs. achievement scores) correlate with social behavior.

### Social behavior and academic achievement

Within the social-emotional learning framework, social behaviors support the social medium of learning (Vygotsky, 1978; Slavin, 1995, 2014; Baroody et al., 2016). Farrington et al. (2012) list social behaviors as one of five critical noncognitive factors that predict success beyond school. Two specific types of behaviors can be linked to academic achievement: prosocial behavior and peer problems. These two behaviors have been linked to various academic skills such as study habits, and classroom behavior, and peer interactions, which in turn affect academic performance. Wentzel (1993, 1998) has repeatedly found a strong link between prosocial behavior and academic achievement. More recently, Gerbino et al. (2018) analyzed data from an Italian large-scale assessment. They demonstrated that prosocial behavior remained a significant predictor of grades even after accounting for other variables such as personality factors and IQ. Relatedly, Lewis et al.'s (2017) large-scale twin study indicated that prosocial behavior substantially improved predications based on genetics and environmental characteristics. Similarly, peer problems also correlate to lower achievement (Wentzel and Caldwell, 1997), and Malecki and Elliot (2002) found that poor social skills indicated worse performance on achievement tests. More recently, Askell-Williams and Lawson (2015) showed that children with peer problems were more likely to have lower academic motivation as well as other school-related difficulties.

Nonetheless, some inconsistent results remain. Adams et al. (1999) found that after accounting for hyperactivity, conduct problems, and emotional problems, neither peer problems nor prosocial behavior related to math achievement test results; however, prosocial behavior remained related to reading achievement test results. This contrasts with Gerbino et al. (2018) results which indicated that prosocial behavior remains a significant correlate of overall grades after accounting for multiple other factors.

### Grades vs. achievement tests

One factor that could help explain such discrepancies is the use of grades vs. achievement tests to measure academic achievement. For instance, many educators include behavior measures in their grading (Cross and Frary, 1999), and grades have been shown to reflect numerous personality factors in addition to academic competence (Borghans et al., 2011; Andrei et al., 2015; Lechner et al., 2017; Gerbino et al., 2018). For example, Lockl et al. (2017) found that theory of mind in kindergarten predicted grades in grade 1 and 2, but they did not examine any connection to achievement test scores. Moreover, theory of mind represents a specific aspect of social development, and more research examining peer problems and prosocial behavior is needed. Despite this, large-scale studies examining both grades and achievement testing alongside social behavior are rare.

### Moderating variables

Among others, two key moderating variables in these studies have been socio-economic status (SES) and gender. Children of higher SES tend to show fewer social problems and more prosocial behavior (Letourneau et al., 2013). They have higher levels of inclusion at school (Veland et al., 2015), receive better grades (Lekholm and Cliffordson, 2008), and perform better on other achievement measures (Sirin, 2005). Furthermore, lower SES children engage in more prosocial behavior (Piff and Robinson, 2017), but they are also at higher risk of developing social problems (Bradley and Corwyn, 2002). Additionally, well established differences have been found in developmental trajectories for boys and girls for prosocial behavior and peer problems (Card et al., 2008; Chaplin and Aldao, 2013), as well as in both math and reading achievement (Robinson and Lubienski, 2011). It is therefore important to consider both gender and SES as important moderators when examining achievement and social behavior.

### The present study

This study investigates the differential effects of prosocial behavior and peer problems on both grades and achievement tests. We examine both math and reading achievement measures in a longitudinal, large-scale assessment, and account for both gender and socioeconomic status (SES). The use of large-scale panel data is important because the effects of social behavior are predicted to be important, but indirect (Farrington et al., 2012). Because such indirect effects are a particularly difficult hurdle when predicting effects of different strengths, we use the NEPS database (Blossfeld et al., 2011), which includes data from a large-scale German longitudinal survey with enough participants to model all necessary variables.

Based on the role of social skills as a noncognitive factor in learning (see Farrington et al., 2012), we expect that more desirable social behavior will correlate to both better grades and better competencies in reading and math. In a recent similar study, internalizing problems were shown to have a detrimental effect on achievement outcomes of secondary students (Deighton et al., 2018). However, because grades are a better reflection of noncognitive factors in learning, our first prediction is that grades will be more impacted by social behaviors than competency (see Borghans et al., 2011; Lechner et al., 2017). Furthermore, both gender and SES are well-known moderators of achievement and social behavior. Therefore, our second prediction is males will do better on math measures while females will do better on reading measures, and that students with higher SES will outperform those with lower SES on both measures. In a similar analysis, (Gerbino et al., 2018) showed that effects of social behaviors on grades remained after accounting for moderating personality factors. Therefore, our final predication is that the effects of prosocial behavior and peer problems will remain after accounting for gender and SES as determined by parental education.

## Methods

### Data and participants

All data came from the NEPS database (Blossfeld et al., 2011), which contains multiple large representative cohorts of German students. NEPS data are collected each year from selected students, teachers, parents, and administrators. We focused on NEPS cohort 3, which began in grade 5. We used data from waves 1 (grade 5, October 2010–January 2011), 2 (grade 6, October 2011–January 2012), and 3 (grade 7, October 2012–January 2013). All participants with data on any of the key variables were included in our models. Because of small differences in who took the reading and math competency NEPS tests and in who reported their grades for German and math, the number of participants varied slightly between both datasets. We provide an overview of the participants in Table 1.

**Table 1:** Participant information.

	**Math model (*n* = 3310)**	**Reading model (*n* = 3308)**
**GENDER (PERCENT)**		
Male	51.6%	50.6%
Female	48.2%	49.4%
**AGE (MEAN, SD)**		
Years	12.0 (0.8)	12.0 (0.8)
**PARENTAL EDUCATION (PERCENT)**		
Basic	14.0%	14.0%
Vocational	56.0%	56.0%
University	30.0%	30.0%
**SCHOOL TYPE (PERCENT)**		
Secondary—Hauptschule	7.6%	7.6%
Secondary—Realschule	22.1%	22.2%
Secondary—Gymnasium	52.2%	52.4%
Other	18.1%	17.8%

*Parental Education was determined by CASMIN*.

### Data collection

We focused on a small subset of the collected data for our models: math competency, math grades, SDQ scores for the subscales of peer problems and prosocial behavior, gender, and parental education level.

#### Competency measures

We used the uncorrected weighted maximum likelihood estimates (WLE) from grades 5 and 7 in the NEPS dataset for both math and reading competency. Analyses by the NEPS team confirmed unidimnsionality, reliability, and measurement invariance of these estimates across gender, books in household, and migration background (Haberkorn et al., 2012; Krannich et al., 2017). Math and Reading competency were assessed in waves one and three (grades five and seven).

#### Grades

Self-reported math and German whole-year grades were used for grades 5 and 7. In the German school system, grades are ordered from 1 to 5, with lower scores representing better grades (1 = very good, 2 = good, 3 = satisfactory, 4 = sufficient, 5 = failing).

#### Prosocial behavior and peer problems

The prosocial behavior and peer problems subdimensions of the Strengths and Difficulties Questionnaire (SDQ) were used to assess social behavior in wave two. The SDQ is a frequently used questionnaire to assess psychological characteristics of children (Goodman, 1997; Goodman et al., 2010) and has been demonstrated to meet basic psychometric properties for longitudinal analyses in German samples (DeVries et al., 2017). The other three SDQ subscales were unavailable in the NEPS database for this time period.

#### Socioeconomic status (SES)

In parent interviews in wave one, a parent responded about his or her own educational attainment as well as his or her partner's attainment. Responses were rated based on the Comparative Analysis of Social Mobility in Industrial Nations (CASMIN) scale (Brauns et al., 2003). The scale was reduced to three basic categories: low (no secondary degree, or secondary degree with basic vocational training), intermediate (advanced vocational training or vocational postsecondary school), and high (university level or higher). Only the higher rating from either parent was used for each child.

### Analysis

**Figure 1:**
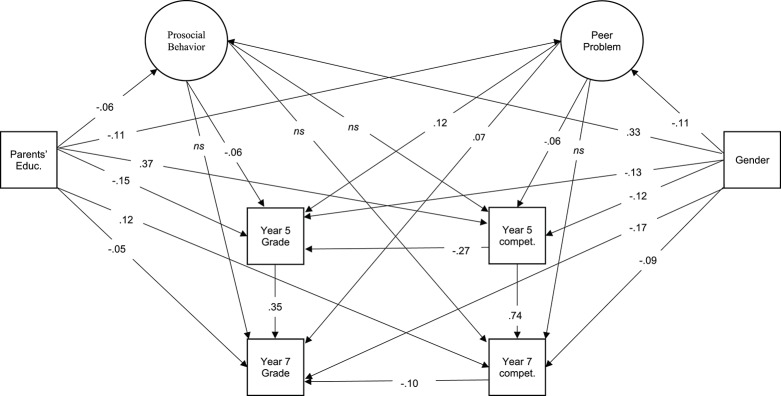
Math Model with Significant Path Loadings. Parents' Educ. refers to parental education level as determined by CASMIN. Compet. refers to uncorrected WLE reported from NEPS competency assessments. Grades refer to final grade in the previous year. Factor loadings of SDQ items for the Prosocial Behavior and Peer Problems scales can be seen in Table 2.

**Figure 2:**
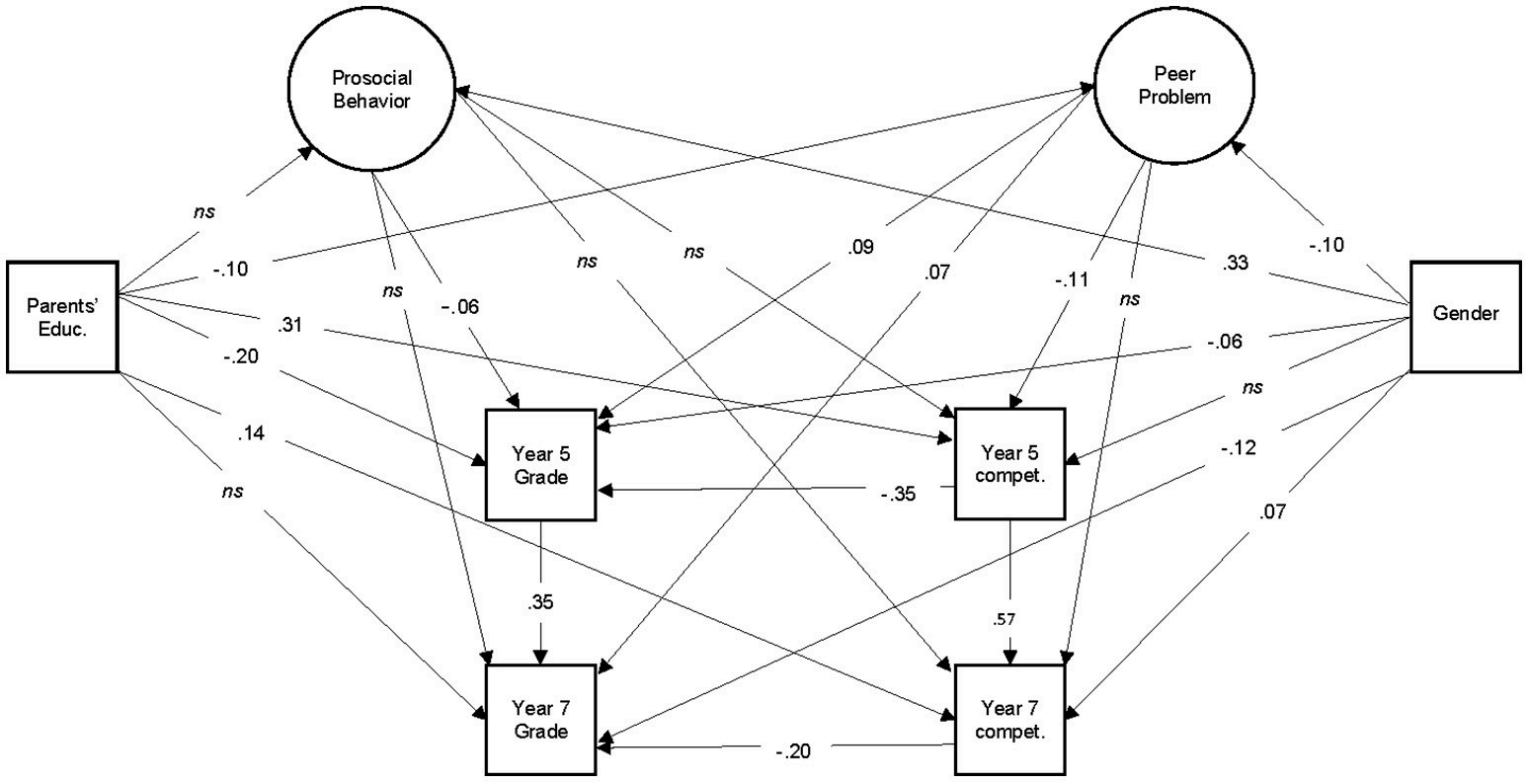
Reading Model with Significant Path Loadings. Parents' Educ. refers to parental education level as determined by CASMIN. Compet. refers to uncorrected WLE reported from NEPS competency assessments. Grades refer to final grade in the previous year. Factor loadings of SDQ items for the Prosocial Behavior and Peer Problems scales can be seen in Table 2.

We analyzed the data with structural equation modeling (SEM). Separate models were calculated for math and reading. A confirmatory factor analysis was conducted for each model with prosocial behavior and peer problems treated as latent variables calculated from individual items from relevant SDQ subscales. Additionally as depicted in Figures 1, 2, gender, parental education, grades (5th and 7th year), and competency were regressed onto each other and the latent variables. Mplus was used for all SEM analyses (Muthén and Muthén, 1998-2017), and an example of our Mplus instruction file is available in the Appendix. Estimations were performed using robust maximum likelihood estimation (MLR), and we report root mean square error of approximation (RMSEA), comparative fit index (CFI) and square root mean residual (SRMR). Acceptable fits included RMSEA < 0.08, CFI > 0.90, and SRMR < 0.10, and good fits included RMSEA < 0.05, CFI > 0.95, and SRMR < 0.08 (Hu and Bentler, 1998).

## Results

### Model fits

#### Math

Overall, the math model produced a good fit of the data, RMSEA = 0.036 (90% CI = 0.033–0.039), CFI = 0.93, and SRMR = 0.040. While the CFI falls below our threshold of 0.05, it remains in the acceptable range. Despite this, the RMSEA and SRMR are well below the threshold for a good fit. We therefore concluded we had a good fit.

#### Reading

Similarly, the reading model provided a good fit, RMSEA = 0.044 (90% CI = 0.038–0.049), CFI = 0.92, and SRMR = 0.046. As in the math model, the CFI was below threshold for a good fit, but was in the range of acceptable fits. Given the good values for the RMSEA and SRMR, we concluded that the fit was good.

#### Reliability and factor loadings for the latent factors

Cronbach's α for peer problems was 0.60, and for prosocial behavior was 0.71, while McDonald's total ω for peer problems was 0.61 and for prosocial behavior was 0.72. Factor loadings for both the math and reading models can be seen in Table 2. They were significant at *p* < 0.001, and ranged between 0.38 at and 0.69. While Cronbach's α and McDonald's ω for the prosocial peer problems were low, overall the measures performed similarly to values from the meta-analysis conducted by Stone et al. (2010). Given the acceptable fit values and overall good model fits, we conclude the models fit the data reasonably well and provided sufficient reliability.

**Table 2:** Standardized factor loadings for peer problems and prosocial behavior.

**SDQ items**	**Math M (SE)**	**Reading M (SE)**
**PEER PROBLEMS**		
Item 3: Loner	0.40 (0.03)	0.38 (0.03)
Item 5: Has Friends	0.45 (0.03)	0.46 (0.03)
Item 6: Popular	0.48 (0.03)	0.46 (0.03)
Item 8: Is teased	0.61 (0.03)	0.59 (0.03)
Item 10: Gets along better with adults than with children	0.45 (0.03)	0.47 (0.03)
**PROSOCIAL BEHAVIOR**		
Item 1: Considerate	0.62 (0.02)	0.62 (0.02)
Item 2: Likes to share things	0.51 (0.02)	0.51 (0.03)
Item 4: Helpful	0.68 (0.02)	0.69 (0.02)
Item 7: Nice to younger children	0.51 (0.02)	0.51 (0.03)
Item 9: Often helps voluntarily	0.53 (0.02)	0.53 (0.02)

*All loadings were significant at p < 0.001. All other loadings and path values for the math and reading models are visible in Figures 1, 2, respectively*.

### General findings of prosocial behavior and peer problems

The standardized path loadings are reported in Figures 1, 2. Prosocial behavior only related to both math and reading grades in grade 5. It did not relate to either math or reading competency. Peer problems, however, were significantly related to math grades at year 5 and 7, as well as competency in grade 5 in both math and reading models.

### Grades vs. achievement scores

As seen in Figures 1, 2, peer problems were predictive of grades broadly in both the reading and math models, and only of competency in the 5th grade. Meanwhile, prosocial behavior was significantly related to 5th year grades, but not 7th year, and never to competency.

We conclude that there is a greater overall relationship between grades and social behavior, particularly peer problems. Although, there is an indication of a relationship between peer problems and competency at an earlier grade.

### SES and gender

Figures 1, 2 also indicate the effects of gender and parental education on competency and grades in both the math and reading models. Parental education was related to better grades and competency in both the math and reading models at both measurement points.

Gender was also a strong predictor of performance. Girls had worse math grades and competency than boys at both measurement points, and they had better grades than boys in both measurement points. However, they had better reading competency than boys in grade 7, but not at grade 5.

Overall, we conclude that gender and SES as determined by parental education correlated significantly with our dependent variables. Loadings from parental education appear to decrease from grades 5 and 7, and the effect of gender on reading became stronger between grades 5 and 7.

### Social behavior on grades after the controlling for moderators

Both of the math and reading models modeled the variance attributed to gender and parental education separately from the variance of prosocial behavior and peer problems. A small to medium sized standardized path loading (path loadings between 0.06 and 0.12) on peer problems on grades and 5th grade competency remained. Thus, we can support our final prediction: that the relationship between social behavior and achievement remains despite including powerful moderating variables in our analyses.

## Discussion

### Overview of findings

Using data from a large-scale assessment of German students in early secondary schools, we provided evidence that social behavior has a disproportionate evidence on grades in comparison to achievement tests. Specifically these findings help reconcile differential findings from studies using only grades or achievement tests as an outcome measure (e.g., (Adams et al., 1999; Malecki and Elliot, 2002; Lockl et al., 2017; Gerbino et al., 2018). In our model, significant relationships between social behavior and both grades and early test scores, but not later test scores, remained. This remained true for both peer problems and prosocial behavior and true in both math and reading models.

### Interpretation and theoretical implication

This novel finding was predicted by previous work which found noncognitive factors correlate more to grades than to IQ scores (Borghans et al., 2011; Lechner et al., 2017). The idea was further developed by Farrington et al. (2012), who identified social skills as one of several types of noncognitive factors influencing grades, one of which was social skills. Moreover, Farrington et al. (2012) called for future research to remedy to major insufficiencies in this line of research: research at the secondary level and research focusing on specific aspects of social skills. Our study addresses both these issues by examining early secondary students and by using the SDQ to define two specific dimensions of social skills: prosocial behavior and peer problems.

We further expand on the findings that internalizing problems are linked to reduced academic performance (Deighton et al., 2018) and that grades are also positively affected by prosocial behavior (Gerbino et al., 2018). One specific aspect of internalizing (i.e., peer problems) had a stronger negative impact on achievement, while prosocial behavior had a smaller positive effect only for grades. We also predicted a significant relationship between achievement test scores and social behavior, but were unable to support this prediction for math or reading beyond the 5th grade. Farrington et al. (2012) argued that social skills had an indirect effect and that it might be stronger for younger learners. Therefore, it is possible that the relationship between social behavior and competency fades as children age, or this relationship is too small to identify at later ages.

### Limitations and future work

Despite our large and robust data set, some limitations remained. Our research focused on 5th and 7th graders. Full data from 9th grade and beyond in this cohort is not yet available. Thus, we cannot yet know the impacts of social behavior and skills on other life success measures and over a longer timeframe. One key assumption from Farrington et al. (2012) is that grades prove to be a better measure of future success, because they include noncognitive factors that are also important in long-term success. Therefore, future longitudinal research is necessary on this and similar cohorts to examine the hypothesis. Furthermore, given only two measurement points, it is difficult to make any causal inferences from this data. Broader longitudinal studies combined with intervention studies and true experiments are required to demonstrate a cause-and-effect connection.

Additionally, our research was further limited by only using limited aspects of social behavior. While prosocial behavior and peer problems are important, other aspects are also important for a full measure of social behavior, such as emotional competence, self-regulation, and aggression. While this data was not fully available in this survey data, future research should endeavor to include additional specific measures of social behavior.

Another limitation comes from the types of data available in the NEPS database. While, the NEPS data-base includes self-reports of grades, it does not include self-reports of SDQ measures. Future work should compare the relationship between other sources of social behavior (e.g., self-report, parent report), and other sources of grades (e.g., teacher reports, academic records, etc.). Another artifact of the NEPS dataset is the order of the data collection. The SDQ subscales were collected between the achievement measures in our study, but we nonetheless treated them as predictors of both earlier and later achievement. Later studies may address this limitation by including more data from later measurement points, as those data become available.

Future work should work to integrate more variables into the analysis. We use a simplified rating of parental education to determine SES; however, parental education represents only a part of the SES, further work should incorporate other measures of SES such as income and living situation into analyses. Additional future work should also incorporate other personality variables, such as compliance, work ethic, and conscientiousness, which may have some overlap with our social behavior measures. Furthermore, the complex interaction of teacher expectation and support based on gender and SES and other variables should be considered. With the integration of these variables alongside an examination of the teacher-student interactions, the reasons for these effects could be further explained.

Lastly, although our dataset was broad and representative, it only included data from students attending schools in Germany. Future research is necessary on datasets from other nations as well as from multinational studies.

### Application for educational practice

Our study further demonstrates the effect of social factors on grades and competency in math and reading. While there may be a potential bias effect on student grades for students based on prosocial behavior, this effect is small. Larger effects were observed for peer problems on both competency and grades. We recommend that teachers be aware of any social problems their students may possess as these learners may require additional support particularly in classrooms that use social learning styles.

## Conclusion

Our goal was to examine the differential impact social behaviors (i.e., peer problems and prosocial behavior) on grades and achievement tests in both math and reading. Our results showed that grades correlate more strongly to social behavior than test scores do at younger ages, and that specifically peer problems have a stronger relationship to academic performance. Researchers should be careful when choosing which measure to use and especially when using both measures interchangeably. Teachers should likewise be aware of the relationship between social behavior and their students' grades. Future research into additional types of social behaviors and skills is necessary to identify the effects of specific aspects of social skills and behavior on specific grade types.

## Ethics statement

This study used existing data from the German National Education Panel Study. From 2008 to 2013, data collection was supervised by the Framework Programme for the Promotion of Empirical Education Research. As of 2014, data collection was carried out by the Leibniz Institute for Educational Trajectories. Because the study used existing data, no new ethical review was required.

## Author contributions

JD served as primary author and data analyst. MG provided writing oversight, feedback, and initial study design, KR provided expertise on developing, modeling, and utilizing control variables (i.e., gender, SES) and theoretical expertise in their implementation in NEPS and their theoretical discussion.

### Conflict of interest statement

The authors declare that the research was conducted in the absence of any commercial or financial relationships that could be construed as a potential conflict of interest.

## Appendix

Mplus instructions for the reading model. The math model was identical, except for substituting math for reading.

Variable:

    Names are tw2_1-tw2_10, gender, rg5, rg7, ParEd, rc5, rc7, SchoolID;

    Usevariables are tw2_1-tw2_10, gender, rg5 rg7 ParEd rc5 rc7;

    missing are all (-99 - -2);

    Cluster is SchoolID;

Model:

    !SDQ subscales

    PP2 by tw2_3 tw2_5 tw2_6 tw2_8 tw2_10;

    PrS2 by tw2_1 tw2_2 tw2_4 tw2_7 tw2_9;

  !Achievement measures on social factors

    rg5 rc5 rg7 rc7 on PP2;

    rg5 rc5 rg7 rc7 on PrS2;

  !Control Variables

PrS2 PP2 rc5 rc7 rg5 rg7 on Gender;

    PrS2 PP2 rc5 rc7 rg5 rg7 on ParEd;

    Gender with ParEd@0;

!Achievement measures - competency predicting grades

    rg5 on rc5;

    rg7 on rc7;

!Achievement measures - Grade 5 to Grade 7 regression

    rg7 on rg5;

    rc7 on rc5;

Analysis:

    type is complex;

    estimator is MLR;

Output:

    stdyx;

    sampstat;

Analysis:

    type is complex;

    estimator is MLR;
